# Ubiquitination of Receptorsomes, Frontline of Plant Immunity

**DOI:** 10.3390/ijms23062937

**Published:** 2022-03-09

**Authors:** Yongming Chen, Yingying Song, Jing Liu, Guangyuan Xu, Daolong Dou

**Affiliations:** 1 MOA Key Lab of Pest Monitoring and Green Management, Department of Plant Pathology, College of Plant Protection, China Agricultural University, Beijing 100193, China; chesy6666@163.com (Y.C.); syymay0519@163.com (Y.S.); liujing01302000@163.com (J.L.); ddou@njau.edu.cn (D.D.); 2 College of Plant Protection, Nanjing Agricultural University, Nanjing 210095, China

**Keywords:** ubiquitination, receptorsomes, posttranslational modifications (PTMs), plant immunity

## Abstract

Sessile plants are constantly exposed to myriads of unfavorable invading organisms with different lifestyles. To survive, plants have evolved plasma membrane-resident pattern recognition receptors (PRRs) and intracellular nucleotide-binding domain leucine-rich repeat receptors (NLRs) to initiate sophisticated downstream immune responses. Ubiquitination serves as one of the most important and prevalent posttranslational modifications (PTMs) to fine-tune plant immune responses. Over the last decade, remarkable progress has been made in delineating the critical roles of ubiquitination in plant immunity. In this review, we highlight recent advances in the understanding of ubiquitination in the modulation of plant immunity, with a particular focus on ubiquitination in the regulation of receptorsomes, and discuss how ubiquitination and other PTMs act in concert to ensure rapid, proper, and robust immune responses.

## 1. Introduction

Sessile plants are constantly exposed to a large diversity of pathogens, including bacteria, fungi, oomycetes, nematodes, and viruses. To survive, plants rely on a two-tiered immune response to fend off pathogens [1,2]. The first layer of the plant immune system is initiated by the recognition of microbe/herbivore-associated molecular patterns (MAMPs/HAMPs), plant-derived endogenous danger-associated molecular patterns (DAMPs), and phytocytokines by PRRs, resulting in PAMP-triggered immunity (PTI) [3,4,5]. Plant PRRs include receptor-like kinases (RLKs) and receptor-like proteins (RLPs) with an ectodomain, a transmembrane domain, and an intracellular kinase domain for RLKs. However, host-adapted pathogens interfere with PTI responses by secreting effectors into the plant cell, resulting in effector-triggered susceptibility (ETS) [6,7]. During their evolutionary arms race with pathogens, plants have evolved a robust resistance response known as effector-triggered immunity (ETI), which is mediated by nucleotide-binding domain leucine-rich repeat proteins (NBS–LRRs or NLRs) that recognize pathogen effectors directly or through effector-induced perturbation [8]. Despite the differences in receptor activation and early signaling mechanisms, the boundaries between PTI and ETI have become increasingly blurred [9]. PTI and ETI trigger a series of overlapping immune responses, including Ca^2+^ influx, reactive oxygen species (ROS) bursts, the activation of mitogen-activated protein kinases (MAPKs), transcriptional reprogramming, and phytohormone production [10]. However, the strength, kinetics, and duration of these events in PTI and ETI show distinct features. Additionally, some central PTI components are required for ETI and vice versa, and these processes mutually potentiate each other [11,12,13,14].

Proper activation of the plant immune system ensures rapid defense responses to protect against potential infections without growth penalty costs. The excessive activation of defense responses can be detrimental, even fatal, to hosts. For instance, over-activation of NLR receptors commonly triggers inadvertent autoimmunity without pathogen attacks in hybrid plants, thereby compromising plant growth [15]. In some extreme cases, autoimmunity is even postembryonically lethal and associated with spontaneous cell death [15,16]. Thus, the activated immune responses should be kept in check to avoid defense from running amok. Ubiquitination, a post-translational modification (PTMs), has emerged as an important regulatory mechanism in modulating immune receptorsome-mediated defense responses [17]. Ubiquitination involves the covalent attachment of ubiquitin, a highly conserved 76-amino-acid protein, to the lysine residues of target proteins. Ubiquitination plays an essential role in regulating nearly all aspects of plant biology, including plant growth, development, and responses to abiotic and biotic stresses. The ubiquitin protein contains seven lysine (K) residues (including K6, K11, K27, K29, K33, K48, and K63) that potentially link to the substrate protein [18]. Among these, K48-linked ubiquitination is the best-studied process and always serves as the prerequisite signal for plant degradation by the 26 S proteasome [18,19,20]. The typical ubiquitination process is composed of three distinct biochemical reactions catalyzed by three different classes of the enzyme, including the ubiquitin-activating enzyme (E1), ubiquitin-conjugating enzyme (E2), and ubiquitin ligase (E3) [19,20,21]. E1 catalyzes ubiquitin activation via adenylation of the ubiquitin C-terminal carboxyl group. The activated ubiquitin is transferred from E1 to the catalytic cysteine residue of E2, which then partners with an E3 ligase to transfer ubiquitin to a lysine of the substrate proteins [20]. The subsequent E1–E2–E3 cascade results in the attachment of a single ubiquitin to the substrate protein, known as monoubiquitination, or leads to the formation of a polyubiquitin chain after the first ubiquitin is linked to the substrate. Further increasing ubiquitin moieties are then linked to the prior ubiquitin molecules through a process referred to as polyubiquitination [18,19,20]. Plant genomes encode a large number of E3 ligases, which are generally classified into three subgroups: the RING (really interesting new gene) and U-box type, the HECT (homologous to E6-associated protein C terminus) type, and the RBR (RING between RING) type [20]. Some of these subgroups were identified to be involved in the regulation of plant immunity-related component turnover, endocytosis, and cytoplasmic transport [21,22,23].

Over the last decade, remarkable progress has been made in understanding the mechanism of receptorsome-mediated plant immunity. In particular, PTM plays an important role in regulating receptorsome activation and attenuation and facilitating effective immune responses. In this review, we focus on the latest progress in the regulation of PRR and NLR signaling by ubiquitination, which is necessary for the appropriate activation of immune signaling and maintaining cellular homeostasis when a threat disappears. We also discuss how ubiquitination and other PTMs act in concert to ensure sophisticated and controllable immune responses.

## 2. Ubiquitination Regulation of Pattern Recognition Receptor Complex

### 2.1. Ubiquitination in Flagellin-Triggered Immune Signaling

**Figure 1:**
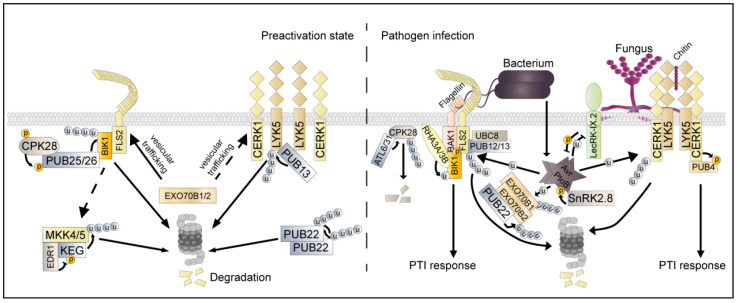
Ubiquitination regulation of PRR receptorsome-mediated signaling in *Arabidopsis*. In the resting state, exocyst subunits EXO70B1/2 regulate trafficking of the bacterial flagellin receptor FLS2 to the plasma membrane (PM). FLS2 associates with non-activated RLCK BIK1 subjected to polyubiquitination regulation by the E3 ligases PUB25/26. CPK28 phosphorylates PUB25/26 and enhances their enzymatic activities. In parallel, the protein abundance of the fungal chitin receptor LYK5 is also regulated by PUB13. The Raf-like MAPKKK family member EDR1 associates with and negatively affects KEG phosphorylation and self-ubiquitination, thereby enhancing MKK4/5 ubiquitination mediated by KEG. In addition, another E3 ligase, PUB22, undergoes degradation by autoubiquitination in a resting state. Upon flagellin perception by its cognate PRR FLS2, BAK1 phosphorylates PUB12/13, which promotes PUB12/13-targeting-activated FLS2, leading to polyubiquitination and degradation. E2 UBC8 partners with PUB13 and mediates FLS2 polyubiquitination. In contrast, chitin induces the dissociation of PUB13 and LYK5, thus reducing LYK5 polyubiquitination and promoting LYK5 protein accumulation. PUB4 is phosphorylated by CERK1 and positively regulates chitin signaling. Unlike the polyubiquitination of BIK1 in a resting state, flg22-induced BIK1 activation requires E3 ligase RH3A/3B-mediated monoubiquitination. In addition, E3 ligases ATL31/6 positively regulate BIK1-mediated immunity by targeting CPK28 for ubiquitination and relief of the CPK28-mediated negative regulation of BIK1. Additionally, PUB22 is stabilized upon flg22 perception by inhibiting the autoubiquitination activity and oligomerization of flg22, which dampens the immune responses by promoting the ubiquitination of EXO70B2 and its degradation via the 26 S proteasome. Bacteria-delivered AvrPtoB ubiquitinates EXO70B1, leading to degradation and thereby suppressing immune responses. In addition, AvrPtoB targets multiple PRRs, including FLS2, CERK1, and LecRK-IX.2, for ubiquitination, thereby suppressing the response of PTI. In turn, PTI activation facilitates LecRK-IX.2 to phosphorylate AvrPtoB, which disrupts the self-association and compromises the virulence of AvrPtoB.

FLS2, one of the most well-studied PRRs, perceives bacterial flagellin (or the epitope flg22) and rapidly recruits BRASSINOSTEROID INSENSITIVE 1-ASSOCIATED KINASE 1 (BAK1) as coreceptor [24,25] and relays the signaling through associated with receptor-like cytoplasmic kinases (RLCKs) [26,27]. PRRs, coreceptors, and RLCKs often form multiprotein PRR complexes known as receptorsomes [17]. The perception of flg22 induces the formation of FLS2 and BAK1, which in turn phosphorylates RLCK BIK1 and thereby leads to BIK1′s dissociation from the FLS2–BAK1 complex; this dissociation then activates diverse downstream signaling events [25,26,27]. The maintenance of an appropriate abundance of FLS2 on the cell membrane surface is critical for flagellin-triggered immune signaling [28,29]. EXO70B1 and EXO70B2, which are exocyst complex subunits and function with soluble N-ethylmaleimide-sensitive factor attachment protein receptor (SNARE) complexes, play key roles in regulating PM-localized FLS2 abundance via membrane-trafficking machinery [28]. Accordingly, the ROS production and callose deposition induced by flg22 were found to be significantly compromised in *exo70B1*, indicating that EXO70B1 and EXO70B2 function as positive regulators in PTI by regulating the trafficking of FLS2 to the PM [28]. Furthermore, EXO70B2 was identified as a target of the E3 ubiquitin ligase PLANT U-BOX22 (PUB22). Interestingly, treatment with flg22 potentially inhibited the autocatalytic ubiquitination activity of PUB22 and resulted in its accumulation, dampening immune responses by mediating the ubiquitination of EXO70B2 and promoting its degradation via the 26 S proteasome (Figure 1) [30,31].

Upon perceiving flagellin, two closely related E3 ligases, PUB12 and PUB13, which interact with and are phosphorylated by BAK1, then are recruited to target the activated FLS2. PUB12/13 then polyubiquitinate the cytosolic domain of FLS2 and promote 26 S proteasome-dependent FLS2 degradation, thereby down-regulating the immunity response (Figure 1) [32,33]. Furthermore, the ARM domain of PUB13 is phosphorylated by BAK1 and generates a dominant negative effect via blocking its ubiquitination activity [32]. UBC8, a member of the group III E2s, was shown to partner with PUB13 to ubiquitinate FLS2 in vitro (Figure 1) [33]. Similarly, tomato SlPUB12 and SlPUB13 work with group III E2s to ubiquitinate and degrade FLS2 [34]. FLS2 activation leads to both ubiquitination and endocytosis, but multiple key sites of FLS2 (which is indispensable for endocytosis) do not affect polyubiquitination for FLS2, indicating that polyubiquitination and endocytosis after FLS2 activation occur through an independent pathway [35]. 

Activation of cytoplasmic RLCK serves as a major event that relays downstream immune signaling [36]. In a resting state, BIK1 constitutively associates with FLS2 at the PM. Protein phosphatase PP2C38 dephosphorylates BIK1 and maintains BIK1 in a non-activated state [37]. Activation of the FLS2–BAK1 complex instead phosphorylates BIK1, further leading to phosphorylation of the plasma-membrane-localized NADPH oxidase RBOHD, which triggers ROS production [38], and phosphorylation of calcium-permeable cyclic nucleotide-gated channels (CNGCs), which triggers calcium influx [39,40]. Recent studies have found that BIK1 homeostasis and activation are regulated by different types of ubiquitination. Two closely related E3 ligases, PUB25 and PUB26, polyubiquitinate BIK1 and control its steady-state levels, thereby negatively regulating plant immunity (Figure 1). In contrast, the monoubiquitination of BIK1 is essential for the activation of BIK1 and the dissociation of BIK1 from the PRR complex [41]. After multiple PAMP treatments, a discrete band of ubiquitinated BIK1 was observed. This BIK1 was found to be different from the ladder-like smear of BIK1 polyubiquitination, suggesting that PAMP perception triggers BIK1 monoubiquitination. Two RING-type E3 ligases, RING-H2 FINGER A3A (RHA3A) and RHA3B, were identified to directly monoubiquinate BIK1, thus enabling endocytosis and signaling activation of BIK1 (Figure 1) [41].

### 2.2. Ubiquitination in Chitin-Triggered Immune Signaling

Chitin, a conserved component of most fungal cell walls, is recognized as a typical fungal MAMP that initiates defense signaling in plants [42]. In *Arabidopsis*, chitin oligomers are recognized by receptors containing the lysin motif (LysM) containing RLKs (LYKs) in the PM. LYK5, a major chitin receptor, shows a high affinity for chitin oligomers. LYK4 also shows a moderate affinity for chitin and shares overlapping functions with LYK5. In line with this result, the *lyk4 lyk5* double mutant was found to completely lose chitin-induced immune responses [43]. Despite neither LYK5 nor LYK4 possessing intracellular kinase activity, another LysM-RLK chitin-elicitor receptor kinase 1 (CERK1, also known as LYK1) functions as an indispensable chitin co-receptor involved in the perception of chitin oligosaccharides and relays the downstream signal via its intracellular serine/threonine kinase activity [44,45]. Chitin oligomers induce heterodimerization between LYK5 and CERK1 and homodimerization of CERK1 (Figure 1). LYK4 also exists in the complex and provides additional chitin affinity and serves as a scaffold protein [42,43]. Intriguingly, BAK1 interacts with CERK1 and phosphorylates CERK1 in the juxtamembrane region under immune elicitation, which renders CERK1 more stable and transfers it into a primed state [46]. The dynamic interactions of co-receptors allow plants to respond to pathogen attacks in a more rapid and efficient way. 

Plasma-membrane-localized CERK1 and LYK5 presented constitutive endomembrane trafficking [47]. Similar to the exocyst complex trafficking of FLS2 to the PM, EXO70B1 and EXO70B2 are also required for immune responses triggered by chitin [31]. CERK1 accumulation at the PM was found to be impaired in *exo70B1-3* [28]. Ubiquitin E3 ligases PUB22/PUB23/PUB24 promote EXO70B2 degradation and thus attenuate chitin responses upon chitin perception [31]. Ubiquitination may combine with the exocyst complex to regulate the endomembrane trafficking of plant immune receptors. Additionally, PUB12 and PUB13 directly interact with LYK5 and CERK1 and meditate their protein abundance [48,49]. PUB13 ubiquitinates the LYK5 kinase domain in vitro and may promote the degradation of LYK5 via the ubiquitin/26 S proteasome pathway in unaffected cells. Consistently, greater protein accumulation of LYK5 was observed in the pub13 mutant compared with the wild type [49]. Unlike the flg22-induced FLS2 degradation induced by PUB12/13, chitin induces the dissociation of PUB13 and LYK5, thereby promoting LYK5 protein accumulation [42,49]. Treatment with chitin also induces LYK5 to relocalize into mobile intracellular vesicles, while the endocytosis of LYK5 is dependent on CERK1 kinase activity [47]. PUB12 and PUB13 interact with the intracellular domain of CERK1 in a kinase-activity-dependent manner, but evidence showing that PUB12/13 directly ubiquitinates CERK1 is still missing [48,49]. Thus, the same E3 ligases could ubiquitinate multiple PRRs and regulate their homeostasis via different mechanisms. In contrast with PUB12/13, PUB22/23/24, and PUB25/26, which negatively regulate PRR signaling, PUB4 is phosphorylated by CERK1 and positively regulates chitin signaling [50,51]. Although PUB4 interacts with CERK1, it remains to be investigated how PUB4 plays a positive role in chitin-triggered signaling and how PUB4 coordinates with PUB12/13-mediated ubiquitination [52]. In parallel, the rice E3 ligase SPL11 targets RLK SPL11 cell-death suppressor 2 (OsSDS2), leading to polyubiquitination and degradation and negatively regulating cell death and plant immunity in rice [53]. Another E3 ligase in rice, XA21 binding protein 3 (OsXB3), positively regulates RLK OsXA21-mediated signaling [54]. It remains unknown whether OsXB3 directly ubiquitinates OsXA21.

## 3. Ubiquitination Regulation of NLR Proteins

### 3.1. Ubiquitin E3 Ligase SAUL1 Serves as a Guardee Monitored by NLR Pairs

**Figure 2:**
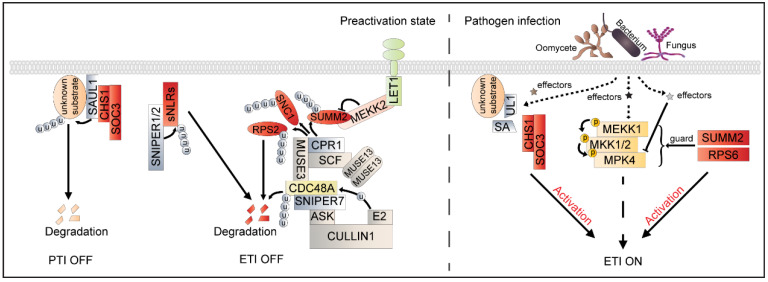
A working model of the ubiquitination regulation of NLR homeostasis. Under normal conditions, a potential unknown substrate is ubiquitinated by the E3 ligase SAUL1, which leads to the degradation of SAUL1′s substrate and deactivates PTI. In addition, SAUL1 associates with the NLR pairs SOC3–CHS1 and is required for SOC3-mediated immune activation. Another E3 ligase, SCFCPR1, interacts with SNC1, RPS2, and SUMM2 and contributes to their ubiquitination and degradation. The TRAF protein MUSE13 homodimerizes and heterodimerizes with NLRs and SCFCPR1 to facilitate SNC1 and RPS2 turnover. The E4 ligase MUSE3 associates with SNC1 and promotes efficient elongation of the ubiquitin chain in NLRs. AAA–ATPase CDC48A interacts with MUSE3 and contributes to the NLR polyubiquitination process. In contrast, the SCFSNIPER7 complex regulates unfoldase CDC48A protein turnover and modulates immune output. MEKK2 scaffolds RLK LET1, which stabilizes SUMM2, and counter-regulates SCFCPR1-mediated SUMM2 ubiquitination. SNIPER1 and its homolog SNIPER2 globally control the protein levels of sNLRs for the immediate attenuation of immune output to effectively avoid autoimmunity. When plants are challenged with an incompatible pathogen carrying an unknown effector that specifically targets SAUL1, SAUL1 is inactivated, leading to the accumulation of the SAUL substrate, which represses PTI. Meanwhile, the inactivation of SAUL1 results in the constitutive activation of SOC3, triggering strong ETI. The effectors induce MEKK1–MKK1/MKK2–MPK4 cascade activation. However, some effectors may disrupt the MEKK1–MKK1/MKK2–MPK4 cascade, leading to CNL SUMM2 and TNL RPS6 activation and intensive activation of the ETI response.

NLRs are tripartite-domain proteins with a variable N-terminus, a central nucleotide-binding and oligomerization domain (NOD), and a C-terminal leucine-rich repeat (LRR) domain. Based on the distinctive features of the N-terminal domain, plant NLRs are classified into three groups: coiled-coil (CC) domain-containing NLRs (CNLs), Toll/interleukin-1 receptor (TIR) domain-containing NLRs (TNLs), and RPW8 domain-containing NLRs (RNLs) [55,56]. In general, CNLs and TNLs are sensor NLRs (sNLRs), which are highly polymorphic and recognize specific effectors. RNLs are referred to as helper NLRs (hNLRs), which function downstream of diverse sNLRs and are evolutionarily more conserved [57]. Improper activation of NLRs is the most prominent factor leading to autoimmunity [15]. For instance, a gain-of-function point mutation in the TNL chilling sensitive 1 (CHS1, also known as TN1) showed constitutive defense responses at low temperatures. It was previously noted that E3 ligases play important roles in regulating NLRs. Overexpression of the E3 ligase SAUL1 (SENESCENCE-ASSOCIATED E3 UBIQUITIN LIGASE 1) leads to an autoimmune phenotype that features constitutive defense-gene expression and enhanced disease resistance, indicating that SAUL1 plays a positive role in immunity [58]. Furthermore, overaccumulation of SAUL1 partially relies on the TNL protein TN2, which shows a head-to-head arrangement with other TNL suppressors of *chs1-2*, 3 (SOC3) in a gene cluster [59]. SOC3 is also consistently required for autoimmunity caused by the overaccumulation of SAUL1 (Figure 2). Additionally, homo- and hetero-dimerization of the TIR domains is required for effector recognition and downstream signal activation in some NLR pairs [60,61]. SOC3 in association with TN2 and SOC3–TN2 might function as NLR heteropairs to guard a yet-unknown substrate of SAUL1 (Figure 2) [59]. In addition, TN2 is responsible for the autoimmunity phenotypes of *exo70B1*, and the overaccumulation of CALCIUM-DEPENDENT PROTEIN KINASE 5 (CPK5) results in TN2-dependent disease resistance [62,63]. However, whether CPK5 is involved in the process of SOC3–TN2 and SOC3–CHS1 NLR pair activation remains to be further studied. Moreover, the SOC3 TIR domain is associated with the mutated form of CHS1 but not the wild type, which might lead to activation of SOC3 and initiate constitutive defense responses under low temperatures [64]. Intriguingly, the loss-of-function of SAUL1 also exhibits cell death and seedling lethality. The apparent contradiction of the over-expression and knock-out of SAUL1 indicates SAUL1′s complicated role in plant immunity. The autoimmune phenotype in SAUL1 mutant plants is dependent on EDS1 and PAD4 but not SAG101 [65]. The EDS1–PAD4 complex is an essential hub for TNL-mediated signaling [66]. Both SOC3 and CHS1 are required for *saul1*-mediated autoimmunity, indicating that SAUL1 can be guarded by multiple TNLs [59]. Notably, CHS1 is not required for the autoimmunity caused by SAUL1 overexpression. In addition to its important role in TNL signaling, SAUL1 functions redundantly with PUB43 to promote PTI signaling [58,67]. The E3 ligase activity of SAUL1 has been identified in vitro, and some potential substrates have been reported, such as CHLOROPLAST-LOCALIZED SENESCENCE-ASSOCIATED PROTEIN (CSAP) and ARABIDOPSIS ALDEHYDE OXIDASE 3 (AAO3) [59,68,69]. However, whether CSAP and AAO3 are involved in plant immunity remains unclear.

Another PM-localized protein, RPM1-INTERACTING PROTEIN4 (RIN4) (a negative regulator in flg22-triggered immunity), is guarded by the CNL proteins RPS2 and RPM1. Recently, RIN4 was shown to be involved in AvrRpm1-induced FLS2 degradation [70,71]. MAPK activation is one of the typical downstream responses in both PTI and ETI signaling [72]. It was reported that MEKK1–MKK1/MKK2–MPK4 is monitored by CNL SUMM2 and TNL RPS6 (Figure 2) [73,74]. In addition to SUMM2, MEKK2 is also required for activation of the defense responses in the *mekk1* mutant and functions upstream of SUMM2 [75]. MEKK2 stabilizes SUMM2 by counter-regulating SUMM2 ubiquitination and degradation (Figure 2) [76].

### 3.2. E3 Ligase-Mediated NLR Ubiquitination and Degradation

In addition to the E3 ligase serving as a guardee in NLR-mediated signaling, E3 ligase-mediated ubiquitination plays an important role in regulating the homeostasis of NLR immune receptors. The E3 ubiquitin ligase Skp1-Cullin1-F-box, with the constitutive PR gene expression 1 (SCF^CPR1^), negatively regulates the stability of NLR immune receptors [18,76]. CPR1 interacts with SNC1 and RPS2, and the overexpression of CPR1 results in degradation of SNC1 and RPS2 via the 26 S proteasome, thereby suppressing the auto-activation of immune responses mediated by these two NLR proteins (Figure 2) [77,78]. *UBC13*, which encodes an E2 ubiquitin-conjugating enzyme required for K63-linked polyubiquitination, interacts with CPR1 but does not affect the SNC1 protein level, indicating that UBC13 is not involved in CPR1-mediated SNC1 protein degradation (Figure 2) [79]. Efficient ubiquitin chain elongation relies on E4 ligase activity [21]. *MUTANT, SNC1-ENHANCING 3* (*MUSE3*), which encodes a ubiquitin E4 ligase, associates with SNC1 and promotes polyubiquitination of NLR proteins (Figure 2) [79,80]. In addition, CELL DIVISION CONTROL PROTEIN 48 (CDC48A), an AAA-ATPase, interacts with MUSE3 and the proteasome regulator PTRE1, thereby contributing to the SNC1 polyubiquitination process and degradation via the 26 S proteasome [81,82]. Moreover, CDC48A interacts with another F-box E3 ligase, SNIPER7 (SNC1-INFLUENCING PLANT E3 LIGASE REVERSE GENETIC 7), while the SCF^SNIPER7^ complex regulates unfoldase CDC48A turnover and modulates immune output (Figure 2) [83]. Two redundant TUMOR NECROSIS FACTOR RECEPTOR-ASSOCIATED FACTOR (TRAF) proteins, MUSE13 and MUSE14, associate with SCF^CPR1^ and form a plant-type TRAFasome, which facilitates SNC1 and RPS2 turnover (Figure 2) [84]. Moreover, degradation of MUSE13 is inhibited by the proteasome inhibitor, indicating that MUSE13 stability is regulated by the 26 S proteasome. The F-box protein SNIPER4 associates with and regulates the turnover of MUSE13 and MUSE14, which may serve to restore SNC1 accumulation [84,85]. A recent report showed that two master E3 ligases, SNIPER1 and its homolog SNIPER2, globally control the protein levels of sNLRs, leading to immediate attenuation of immune output and effectively avoiding autoimmunity (Figure 2). This broad regulation is achieved via direct recognition and ubiquitination of the NB domains of sNLRs. Additionally, SNIPER1 has no effect on hNLR-mediated autoimmunity. Therefore, SNIPER1 and SNIPER2 seem to broadly control the protein levels of sNLRs but not hNLRs [86]. 

*SIDEKICK SNC1 1* (*SIKIC1*), *SIKIC2,* and *SIKIC3*, three redundant NLRs arranged in tandem in the *RPP4*-*SNC1* cluster, are also responsible for SNC1-mediated defense responses [87]. Furthermore, the protein level of SIKIC2 is under the control of two previously uncharacterized redundant E3 ubiquitin ligases: MUSE1 and MUSE2 [87]. Taken together, NLR homeostasis is mainly regulated via ubiquitination mediated by E3 ligase for degradation. However, it remains unknown how these shared E3 ligases maintain specificity in regulating different instances of NLR-mediated immune signaling.

Emerging evidence indicates that NLR protein levels are regulated by multiple dimensions. MUTANT, SNC1-ENHANCING, 7 (MUSE7), an evolutionarily conserved putative kinase substrate, negatively regulates the protein accumulation of NLRs, including SNC1, RPS2, and RPM1. However, no interactions were detected between MUSE7 and CPR1 or HSP90.3 [88], suggesting that MUSE7 may affect NLR protein biosynthesis or degradation through uncharacterized proteins [88]. HSP90 also associates with RAR1 and SGT1, thereby regulating the correct folding, assembly, and homeostasis of NLRs [89]. Point mutations in the two HSP90 genes HSP90.2 and HSP90.3 consistently lead to the increased accumulation of NLR immune receptors SNC1, RPS2, and RPS4 [90]. HSP90 interacts with SGT1 to chaperone its clients to the SCF complex, suggesting that the HSP90–SGT1 chaperone complex is involved in the formation of SCF E3 ubiquitin ligase complexes that target NLRs for degradation [90]. The N-terminal of SNC1 is acetylated by two different N-terminal acetyltransferase (Nat) complexes (NatA and NatB) that regulate the accumulation of SNC1. Nat complex A (NatA)-mediated acetylation serves as a degradation signal, while NatB-mediated acetylation stabilizes the SNC1 protein and increases plant immunity [91]. Acetylation and ubiquitination appear to simultaneously contribute to NLR stability control in plant immunity, but how acetylation and ubiquitination cross-regulate NLR degradation remains to be determined.

## 4. Interplay between Ubiquitination and Other PTMs

### 4.1. Intertwined Regulation of Plant Immunity via Ubiquitination and Phosphorylation

As the most representative and well-studied PTM, ubiquitination intertwined with phosphorylation regulates the life cycle and physical activity of the substrate, which is a common process that orchestrates plant immunity, particularly in regulating PRR complex homeostasis and activation. BAK1, but not FLS2, phosphorylates PUB12/13, which boosts the association of PUB12/13 and FLS2 and promotes FLS2 ubiquitination and degradation [33]. However, it remains unknown whether the phosphorylation of PUB12/13 mediated by BAK1 affects their respective E3 ligase activities. Another RLK, BRASSINOSTEROID INSENSITIVE1 (BRI1), phosphorylates PUB12/13 and enhances their E3 ligase activities, thereby promoting BRI1 ubiquitination and degradation [92]. Similarly, rice RLK OsSDS2 phosphorylates OsSPL11, which in turn ubiquitinates OsSDS2, leading to degradation in a kinase-dependent manner, suggesting that the phosphorylation of OsSPL11 by OsSDS2 may promote the E3 enzymatic activity of OsSPL11. Thus, phosphorylation appears to regulate ubiquitination by enhancing the interactions between the E3 ligase and substrate or enhancing E3 ligase activities to promote the ubiquitination process. BIK1 serves as a convergence signaling node whose homeostasis is critical for the balance of growth and immunity in plants. The E3 ligase activities of PUB25 and PUB26 responsible for BIK1 stability are stimulated by CPK28-mediated phosphorylation. Although CPK28 is associated with PUB25/26 independent of flg22 treatment, increased CPK28 kinase activity following flg22 treatment further destabilizes BIK1 [93]. Interestingly, flg22 treatment enhanced another pair of closely related ubiquitin ligases, ARABIDOPSIS TOXICOS EN LEVADURA 6 (ATL6) and ATL31 associated with CPK28 in the PM, further mediating CPK28 degradation (Figure 1). ATL31/6 positively regulates immune responses through the ubiquitination of CPK28 and relieving the CPK28-mediated negative regulation of BIK1 (Figure 1) [94]. Additionally, CPK28 undergoes intermolecular autophosphorylation on Ser318, which is required for the activation of CPK28 under low intracellular [Ca^2+^] to prevent the initiation of an immune response in the absence of infection [95]. Whether this residual phosphorylation status affects the polyubiquitination of CPK28 remains to be determined. Unlike the polyubiquitination of non-phosphorylated BIK1, ligand-induced BIK1 phosphorylation is a prerequisite for RHA3A/B-mediated BIK1 monoubiquitylation, which triggers the release of BIK1 from PRR complexes and endocytic trafficking (Figure 1) [41,96]. It remains to be determined whether BIK1 phosphorylates RHA3A/B and regulates their activities. Notably, BIK1 monoubiquitylation does not affect the kinase activities of RHA3A/B [41].

The strict phosphorylation and ubiquitination regulation of MAPK activation and ROS burst represent a key step for plants to ward off infection. There are two parallel MAPK cascades, MEKK1–MKK1/2–MPK4 and MKKK3/5–MKK4/5–MPK3/6, that serve as the most typical downstream signals in plant immunity. ENHANCED DISEASE RESISTANCE1 (EDR1), a raf-like MAPKKK family member, was reported to be a key negative regulator in plant defense responses by affecting the protein levels of two defense-related MAPKK subfamily members, MKK4 and MKK5 [97]. It was shown that the E3 ligase KEEP ON GOING (KEG) recruits the EDR1 protein to the trans-Golgi network/early endosome vesicles (TGN/EE), which function together to regulate endocytic trafficking and/or the formation of signaling complexes on TGN/EE vesicles during stress responses [98]. Recently, Gao et al. provided direct evidence that KEG ubiquitinates and mediates the degradation of MKK4 and MKK5. KEG phosphorylation and abundance are negatively regulated by EDR1 [99]. EDR1 also interacts with another E3 ligase, ATL1, and negatively regulates its activity (Figure 1) [100]. However, whether EDR1 is involved in the regulation of ATL1 protein phosphorylation status remains to be determined [100]. The phosphorylation of PUB22 by MPK3, which is activated during PTI signaling, reduces the dimerization of PUB22, which increases PUB22 stability and thereby dampens immune responses. It also appears that phosphorylation can regulate E3 ligase abundance by affecting the self-ubiquitination of E3 ligases. Disruption of another activated MEKK1–MKK1/2–MPK4 cascade activates NLR protein SUMM2-mediated autoimmunity via MEKK2, leading to cell death [101]. MEKK2 functions as a negative regulator of MAP kinases. MEKK2 binds to MPK4 and directly inhibits the phosphorylation of MPK4 via upstream MKKs [102]. Moreover, MEKK2 stabilizes LETUM1 (LET1) and SUMM2 and counter-regulates F-box protein CPR1-mediated SUMM2 ubiquitination and degradation (Figure 1) [76]. The MEKK2 kinase mutant (MEKK2^KM^) can still stabilize LET1 and SUMM2, as well as enhancing cell death triggered by SUMM2, indicating that MEKK2 likely serves as a scaffold protein, rather than a kinase, to stabilize the SUMM2 and LET1 protein complex that activates autoimmunity [76]. Moreover, the BIK1 homolog PBL13 phosphorylates and regulates the stability and activity of NADPH oxidase RBOHD-mediated ROS bursts [103]. PBL13-induced RBOHD degradation is likely mediated by PBL13 INTERACTING RING DOMAIN E3 LIGASE (PIRE), which ubiquitinates RBOHD [103]. The phosphomimetic mutant of RBOHD displays enhanced ubiquitination and reduced protein abundance, indicating that ROBHOD stability is coordinately regulated by phosphorylation and ubiquitination.

In light of the importance of ubiquitination in plant immunity, pathogens deploy effectors that exhibit E3 ligase activities to suppress plant defense via the ubiquitination of vital immune components. For example, AvrPtoB possesses E3 ligase activity and suppresses plant immunity through targeting multiple PRRs, including FLS2 and CERK1, for ubiquitination and degradation (Figure 1). In tomato, AvrPtoB ubiquitinates and facilitates the degradation of kinase Fen in the 26S proteasome-dependent pathway, thereby undermining Fen-mediated immunity, while Pto, a paralog of Fen, is recalcitrant to AvrPtoB-mediated ubiquitination. Pto phosphorylates AvrPtoB T450 residue, which inactivates E3 ligase activity [104]. AvrPtoB self-association appears to be essential for this effector’s E3 ligase activity. *Arabidopsis* Lectin RLK LecRK-IX.2 phosphorylates AvrPtoB S335 residue and disrupts the self-association of AvrPtoB (Figure 1). Accordingly, phosphomimetic AvrPtoB S335D fails to ubiquitinate LecRK-IX.2 efficiently, leading to the compromised virulence of AvrPtoB [105]. Whether AvrPtoB T450 is essential for self-association remains unclear. Possibly, multiple plant kinases modify pathogen effectors to dampen their virulence [105]. In contrast, another host kinase, SnRK2.8, is required for AvrPtoB’s toxic functions (Figure 1). SnRK2.8 phosphorylates AvrPtoB, which is required for AvrPtoB’s ubiquitination of FLS2/NPR1 and the inhibition of FLS2–BAK1 complex formation. However, whether the SnRK2.8-mediated phosphorylation of AvrPtoB affects the E3 ligase activity of AvrPtoB is still unclear [106,107]. Moreover, whether the manipulation of numerous pathogen effector activities by host components, such as conserved SNRK–CDPK via phosphorylation, is a common occurrence remains to be defined.

### 4.2. Coordinated Regulation of Plant Immunity by ADP-Ribosylation and K63-Linked Ubiquitination

Like ubiquitination, protein ADP-ribosylation is a reversible and multifaceted PTM that regulates protein activity, stability, localization, and interactions. Protein poly(ADP-ribosyl)ation (PARylation) is primarily catalyzed by poly(ADP-ribose) polymerases (PARPs), which transfer ADP-ribose from nicotinamide adenine dinucleotide (NAD^+^) onto acceptor proteins [108,109]. The covalently attached PAR can be removed by poly(ADP-ribose) glycohydrolase (PARG) from the modified targets [109]. In plants, PARylation modification is involved in DNA damage repair and stress responses [108,110]. Among the three PARPs in *Arabidopsis*, PARP1/2 possess PAR polymerase activity, and the activity of PARP2 is enhanced by MAMP treatment and is a major contributor in plant immunity [111,112]. One substrate of PARP2, the forkhead-associated domain protein named DAWDLE (DDL), was identified in a protein array coupled with an in vitro PARylation assay. The PARP2-mediated PARylation of DDL is required for the role of DDL in plant immunity.

Yao et al. recently provided evidence that the E2 ubiquitin-conjugating enzymes 13 A and 13 B (UBC13A/B) are potential substrates of PARP and PARG [108]. Immune activation enhances UBC13B’s association with PARP1 and PAPR2, as well as PARG1 and PARG2. Furthermore, the polyribosylation modification of UBC13B is required for UBC13B to function in plant immunity. In addition, the total K63-linked ubiquitination level in the *parp1/2* mutant was found to be decreased compared with the wild type after flg22 treatment, indicating that PARylation promotes K63-linked ubiquitination, likely by affecting UBC13B’s activity. Furthermore, PARP interacts with protein disulfide isomerases (PDIs), which are involved in the folding of secreted proteins in the endoplasmic reticulum and are likely modified by K63 polyubiquitin chains. However, the ways in which PARylation and ubiquitination act together to regulate protein secretion remain elusive. 

Similar to ubiquitination, proteins subjected to ADP-ribosylation can undergo mono(ADP-ribosyl)ation (MARylation), catalyzed by mono(ADP-ribosyl)transferases (mono-ARTs). Little is known about plant mono-ARTs. Very recently, a study by Kong et al. found a plant-specific noncanonical ADP-ribosyltransferase SRO2 that is involved in the MARylation of immune regulators. Salt-inducible zinc finger 1 (SZF1) and SZF2, both Tandem CCCH Zinc Finger (TZF) family proteins (TZF11 and TZF10, respectively), are key regulators of immune genes expression. SZF1 was identified in a yeast two-hybrid screen for PARG1-interacting proteins. Immune elicitation promotes the ADP-ribosylation of SZF1/SZF2, which can be removed by PARG1. Surprisingly, PARPs do not contribute to the ADP-ribosylation of SZF1 [109], and SZF1/SZF2 undergo MARylation mediated by SRO2 [109]. Importantly, upon immune activation, the MARylation of SZF1 antagonizes the polyubiquitination of SZF1 mediated by the SH3 domain-containing proteins SH3P1/2, thereby stabilizing the SZF1 protein and ensuring proper activation of the immune response [109]. The next question is how the MARylation of SZF1 reduces SZF1’s ubiquitination.

## 5. Conclusions and Perspectives

Ubiquitination, one of the most prevalent PTMs, is of great importance for regulating various signaling components in plant innate immunity. Tight spatiotemporal ubiquitination regulation is paramount for the accurate development of plant immunity [19]. Receptorsomes, key soldiers in the frontline of plant immunity, are subjected to fine-tuning ubiquitination regulation. 

It is now well accepted that E3 ubiquitin ligases from different subfamilies are central to both the PRR complex and NLRs. To date, the ubiquitination of key regulators and signaling components by conventional degradative ubiquitination, such as K48-linked ubiquitination, has been dominant in plant immunity. However, in-depth research is still needed to learn about unconventional and non-degradative ubiquitination. Although much progress has been made in uncovering the E3 ligases involved in plant immunity, the ongoing identification and characterization of novel E3 ligases and their cognate substrate proteins will certainly advance our understanding of plant immunity networks. Growing evidence indicates that multiple pathogen effectors suppress plant immune responses by hijacking plant ubiquitination components, especially E3 ligases. Therefore, re-designing the protein structures in E3 ligases using genome editing tools may also help reshape plant–pathogen interactions and help improve crop resistance to pathogens. Emerging evidence shows that ADP ribosylation plays a vital role in plant immunity. Unsurprisingly, this process may contribute to receptorsome regulation. It would be useful to investigate this hypothesis in future work. 

The interplay between ubiquitination and other PTM definitively increased the functional diversity in the cellular process and enabled plants to respond rapidly and accurately to pathogen attacks. Notably, although ubiquitination is increasingly investigated in plant immunity, the understanding of crosstalk between ubiquitination and other PTMs remains in its early stages. In particular, information is scarce on how the crosstalk between ubiquitination and SUMOylation, acetylation, and glycosylation regulates plant immunity. Phosphorylation- and ubiquitination-mediated regulation in PRR complexes has been extensively studied, but how these two processes cooperatively modulate NLR proteins is much less understood. Moreover, considering the reversibility of many PTMs, the question of whether reverse modification (such as dephosphorylation and deubiquitinating) also orchestrates plant immunity remains to be addressed. 

In addition, the development of proteomic instruments with increased sensitivity, resolution, and mass accuracy will facilitate the comprehensive analysis of ubiquitination and other PTMs. Recently, mass spectrometry (MS)-based proteomics in *Arabidopsis* revealed extensive and complex protein ubiquitination patterns upon immune elicitation, detecting novel proteins for potential ubiquitination associated with plant immunity [113]. Technical breakthroughs will help us identify PTM crosstalk and enable us to better understand the regulation of receptorsomes by ubiquitination and other PTMs.

## Data Availability

Not applicable.
